# Developing a Web-Based Weight Management Program for Childhood Cancer Survivors: Rationale and Methods

**DOI:** 10.2196/resprot.6381

**Published:** 2016-11-18

**Authors:** Fang Fang Zhang, Susan Meagher, Michael Scheurer, Sara Folta, Emily Finnan, Kerry Criss, Christina Economos, ZoAnn Dreyer, Michael Kelly

**Affiliations:** ^1^ Friedman School of Nutrition Science and Policy Tufts University Boston, MA United States; ^2^ Tufts Medical Center Boston, MA United States; ^3^ Baylor College of Medicine Houston, TX United States; ^4^ Tufts University Boston, MA United States

**Keywords:** weight management, childhood cancer survivors, obesity, Web-based, development, nutrition, physical activity

## Abstract

**Background:**

Due to advances in the field of oncology, survival rates for children with cancer have improved significantly. However, these childhood cancer survivors are at a higher risk for obesity and cardiovascular diseases and for developing these conditions at an earlier age.

**Objective:**

In this paper, we describe the rationale, conceptual framework, development process, novel components, and delivery plan of a behavioral intervention program for preventing unhealthy weight gain in survivors of childhood acute lymphoblastic leukemia (ALL).

**Methods:**

A Web-based program, the Healthy Eating and Active Living (HEAL) program, was designed by a multidisciplinary team of researchers who first identified behaviors that are appropriate targets for weight management in childhood ALL survivors and subsequently developed the intervention components, following core behavioral change strategies grounded in social cognitive and self-determination theories.

**Results:**

The Web-based HEAL curriculum has 12 weekly self-guided sessions to increase parents’ awareness of the potential impact of cancer treatment on weight and lifestyle habits and the importance of weight management in survivors’ long-term health. It empowers parents with knowledge and skills on parenting, nutrition, and physical activity to help them facilitate healthy eating and active living soon after the child completes intensive cancer treatment. Based on social cognitive theory, the program is designed to increase behavioral skills (goal-setting, self-monitoring, and problem-solving) and self-efficacy and to provide positive reinforcement to sustain behavioral change.

**Conclusions:**

Lifestyle interventions are a priority for preventing the early onset of obesity and cardiovascular risk factors in childhood cancer survivors. Intervention programs need to meet survivors’ targeted behavioral needs, address specific barriers, and capture a sensitive window for behavioral change. In addition, they should be convenient, cost-effective and scalable. Future studies are needed to evaluate the feasibility of introducing weight management early in cancer care and the efficacy of early weight management on survivors’ health outcomes.

## Introduction

Dramatic improvements in the diagnosis and treatment of cancer in childhood have led to a rapidly growing cohort of survivors, now estimated to exceed 450,000 in the United States [1]. However, this success is associated with the recognition that childhood cancer survivors have significantly elevated risks of premature mortality and serious morbidity [2,3]. Recent studies have shown that childhood cancer survivors not only have significantly higher body mass index (BMI) than their peers [2] but also experience unhealthy weight gain early in treatment, and increases in weight are sustained throughout treatment and beyond [3-5]. Obesity is an established risk factor for cardiovascular diseases (CVD). Childhood cancer survivors are 7 times more likely to die of cardiac causes than the general population [6,7]. They also develop dyslipidemia, hypertension, and insulin resistance or diabetes at a much younger age [8]. Lifestyle interventions are clearly a priority for preventing the early onset of obesity and associated cardiometabolic conditions in this population.

**Figure 1:**
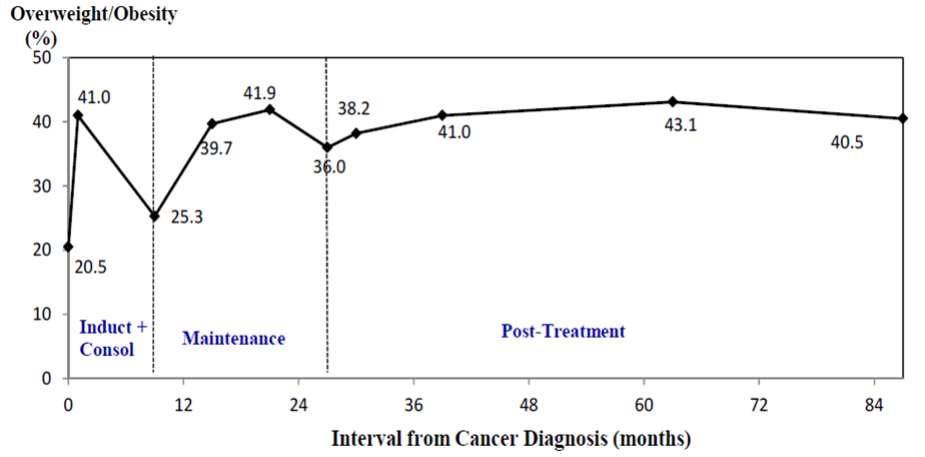
Change in prevalence of overweight and obesity during and after treatment in childhood acute lymphoblastic leukemia survivors (reproduced with permission from Pediatric Blood & Cancer [7]).

Few interventions are designed to promote lifestyle modifications in childhood cancer survivors to reduce obesity and CVD risk [9]. To our knowledge, none have focused on initiating interventions soon after the survivors complete intensive cancer treatment to prevent the early onset of obesity and CVD morbidities. Strong evidence supports that unhealthy weight gain and development of CVD risk factors occur early in treatment and persist beyond treatment completion. For example, in a retrospective cohort of 83 patients of childhood acute lymphoblastic leukemia (ALL) at Tufts Medical Center, we found the percentage of children who were overweight or obese increased from 20% at diagnosis to approximately 36% at the end of intensive cancer treatment (ie, induction and consolidation phases of the treatment, which last between 7 and 9 months for most of the treatment protocols) (see Figure 1). After patients started maintenance chemotherapy (ie, maintenance phase of the treatment, which lasts approximately 18-24 months), the percentage of being overweight or obese increased to 40% at 6 months into maintenance and persisted beyond treatment completion [5]. The early onset of obesity is similarly observed in other studies [3,4] and in a meta-analysis from 21 studies that assessed longitudinal trend of weight patterns in pediatric ALL survivors [10].

Although cancer treatment can directly impact weight patterns, children also develop adaptive behaviors such as poor eating habits and physical inactivity during treatment. We conducted preliminary studies that identified intake patterns and levels of total energy expenditure in young survivors of pediatric ALL and lymphoma [11,12]. These intake and activity patterns were originally thought to be adaptive responses to cancer treatment but tend to last beyond treatment completion and are particularly difficult to reverse in long-term survivors. For example, prior studies have provided consistent evidence that childhood cancer survivors are less active than their peers [13,14]. Our study in survivors of childhood ALL and lymphoma found that the mean level of total energy expenditure (2073 kcal/day) was nearly 500 kcals lower than the estimated energy requirement, supporting the need of increasing physical activity to fill this gap [11]. Such a large gap is unlikely to be reversed by exercise alone. Our prior study [12] and those of others [15-17] have also shown that childhood cancer survivors have poor adherence to existing dietary guidelines. Their intake patterns are particularly low in fiber and whole grains and high in sodium and empty calories (calories from solid fats and added sugars), all of which are established risk factors for obesity and CVD-related morbidities. Further, family environment plays an important role in shaping children’s dietary and activity behaviors [18-23], and parenting style and practices can be particularly important for children diagnosed with cancer at a young age [24] (eg, the peak age of ALL diagnosis in children is 2-5 years old [25]). As reported in qualitative research, parents practice permissive parenting related to unhealthy eating and sedentary behavior while the child is going through cancer treatment, which they find difficult to reverse following treatment completion [26]. Survivors’ poor intake patterns and sedentary behaviors, which are closely related to parenting style and practices in young survivors, are established risk factors for obesity and cardiometabolic conditions.

Based on these findings, we developed a Web-based weight management program, the Healthy Eating and Active Living (HEAL) program, tailored for families with patients and survivors of childhood ALL, the most common cancer in children. Here we describe the conceptual framework, development process, novel components, and delivery plan of the HEAL program.

## Methods

### Overview

**Figure 2:**
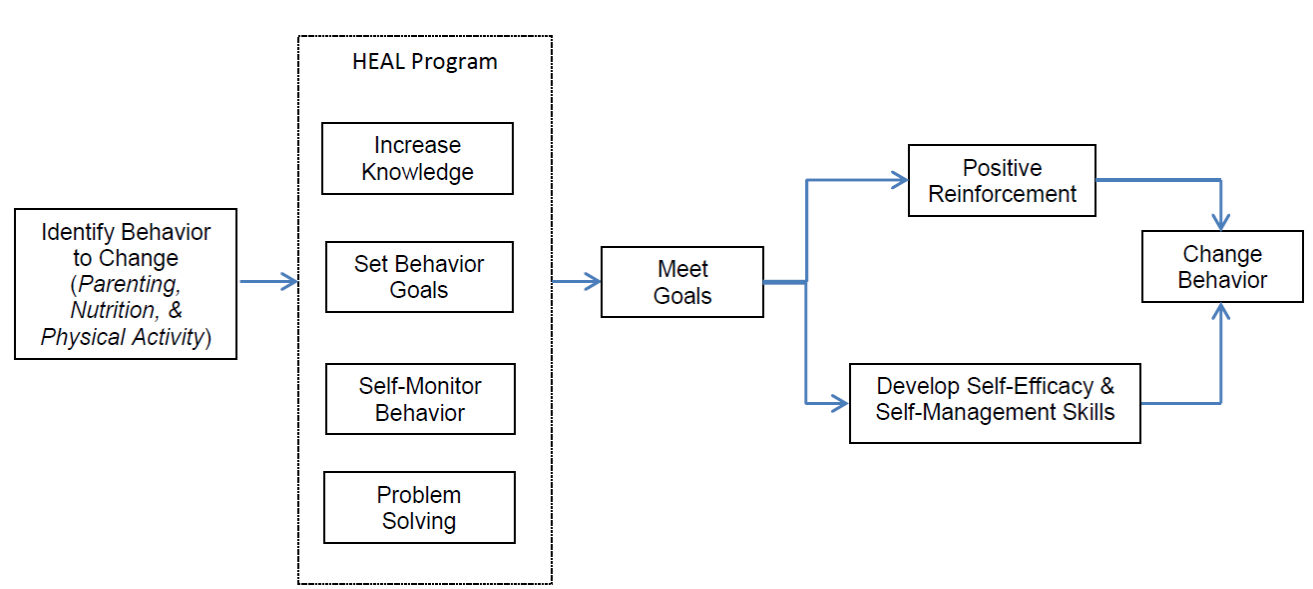
Conceptual framework of the Healthy Eating and Active Living program for childhood cancer survivors.

A multidisciplinary team of researchers (nutritional epidemiologist, pediatric oncologist, nutrition scientist, pediatric dietitian, behavioral scientist, and clinical psychologist) met weekly and identified behaviors that are appropriate targets for weight management in childhood cancer survivors and formulated the intervention components. Guided by strong evidence in childhood cancer survivors and informed by obesity prevention programs in the general pediatric population, we identified parenting, nutrition, and physical activity as 3 behaviors to change for the development of the HEAL program. Following the identification of behaviors to change, we used a basic framework of core behavioral change strategies as a foundation to formulate program goals and components. These core behavioral change strategies are grounded in theoretical models (eg, social cognitive and self-determination theories) [27,28], including increasing knowledge, developing behavioral skills (goal-setting, self-monitoring, and problem-solving), increasing self-efficacy, and providing positive reinforcement (Figure 2) [26].

### Increasing Knowledge

Parental perception of their child’s weight status is a key factor in determining parents’ readiness for weight management for their child [29]. The curriculum therefore starts with increasing knowledge on the patterns of weight gain and the importance of healthy lifestyles in long-term health of childhood ALL survivors, as well as information on how cancer diagnosis and treatment may impact survivors’ weight, eating habits, and physical activity. Following this introduction, the curriculum focuses on 3 areas:

- The parenting focus is to improve parenting practices that facilitate healthy eating and active living in the family. The HEAL program engages parents as the agent of change, because the program targets on young children with ALL who largely fall into the age range of 4 to 10 years old when they start maintenance therapy or within 2 years of treatment completion. It emphasizes parents’ roles in transitioning the family toward healthy eating and active living through modifying the family environment. It also addresses common barriers that parents face in implementing change at the family level.
- The nutrition focus is to enhance diet quality by (1) limiting consumption of sugar-sweetened beverages (SSBs) and foods high in empty calories; (2) limiting consumption of processed foods and snacks high in sodium; and (3) increasing consumption of vegetables, fruits, and whole grains. Although not a direct target for weight management, the program also includes (4) increasing dietary sources of vitamin D and calcium.
- The physical activity focus is to increase activity by (1) reducing screening time, (2) gradually increasing activity to 60 or more minutes/day and incorporating physical activity into daily activities, and (3) incorporating bone-strengthening activity 3 or more days/week [30,31].

### Developing Behavioral Skills

The HEAL program is designed to increase behavioral skills through goal-setting, self-monitoring, and problem-solving. Goal-setting uses SMART planning to help parents set Specific, Measurable, Achievable, Relevant, and Time-bound goals and action items [32]. Parents are asked to set individual goals and action plans within the context of the overall HEAL program goals for parenting, nutrition, and physical activity. Table 1 describes the program goals. For self-monitoring, parents complete weekly online food and activity logs for their child and are provided with the opportunity to monitor progress online. Problem-solving is incorporated to address specific barriers experienced by childhood cancer survivors and families for making healthy food choices or being physical active. These sessions include overcoming food cravings, coping with food aversion and changes in taste preference, fatigue, curbing emotional eating, safety concern for physical activity, and stress and time management. The program also embeds a session that asks parents to identify barriers, list options to overcome barriers, and make plans for realizing options.

**Table 1:** Healthy Eating and Active Living program goals.

Goal	Action plan
Raise obesity awareness	Be aware of the importance of unhealthy weight gain in survivors’ long-term health and how cancer treatment impacts eating, activity, and weight patterns.
Improve parenting style and practices	Increase authoritative parenting, establish healthy routines, offer choices, set expectations, reinforce positive behavior, improve family communication.
Reduce empty calories	Limit consumption of sugar-sweetened beverages and snacks and desserts high in empty calories to 1 or fewer servings/day.
Reduce sodium	Limit eating out at fast food restaurants to 1 or fewer times/week; choose low-sodium option.
Increase fruits, vegetables, and whole grains	Increase fruits and vegetables to 5 or more servings/day; make half of the grains whole grains.
Decrease sedentary behavior	Reduce recreational screen time such as TV, computer/tablet, and video games to fewer than 2 hours/day.
Increase physical activity	Gradually increase physical activity to 60 or more minutes/day
Increase bone-strengthening activity	Gradually increase bone-strengthening activity to 3 or more days/week
Set healthy home environment	Increase family meals to 3 or more times/week; Active together as a family to 3 or more times/week

### Increasing Self-Efficacy

The HEAL program is designed to incrementally support and increase behavioral skills. As these are developed, participants acquire mastery experiences to increase self-efficacy.

### Positive Reinforcement

The program uses positive reinforcement strategies to further promote self-efficacy by sending parents motivational messaging throughout the course of the program. The motivational messages were developed by the clinical psychologist and behavioral scientist on the research team. They serve to remind parents to set goals, track child’s food intake and physical activity, and complete self-guided curriculum sessions and to provide recognition and encouragement after parents complete these activities. Some of the messages are also designed to promote 3 basic psychological needs: competence, autonomy, and relatedness, grounded in self-determination theory (SDT). The motivational messages are sent automatically to parents’ email or through short message service (SMS) after parents enroll into the program, following predetermined algorithms based on program usage and completion status. Alternatively, motivational messages can be delivered by a lifestyle coach trained in motivational interviewing who can further provide feedback based on usage and completion. The use of a lifestyle coach as human contact to support the program is optional and is currently only available in research settings. Parents cannot opt out of motivational messages but can choose to receive messages through email or SMS. Some examples of motivational messages are presented in Table 2. The program also provides multiple digital rewards to reinforce attendance and encourage adherence.

**Table 2:** Examples of Healthy Eating and Active Living program motivational messages.

Type of messages	Examples
To remind parents to complete self-guided curriculum	“Welcome to Session 8! This week is about getting up and getting active. Encourage your child to do physical activity. Any activity counts! Click here to learn more.”
To reinforce completion of self-guided sessions	“Congratulations! You have just completed Session 3. One step closer toward achieving your goal!”
SDT^a^-grounded motivational message promoting relatedness	“Try to get the whole family moving! Believe it or not, your child is watching, and your habits, both good and bad, have a strong influence on them.”
SDT-grounded motivational message promoting competence	“As a parent, you are capable of using positive family communication to talk to your child about eating healthy and being active! Use RECIPE^b^ strategies. Success is yours!”
SDT-grounded motivational message promoting autonomy	“Eat family meals together more often. How often? You decide. You are in charge!”

^a^SDT: self-determination theory.

^b^RECIPE: reflective listening, encouragement, compromise and cooperation, “I” message, practice, and engagement.

## Results

**Figure 3:**
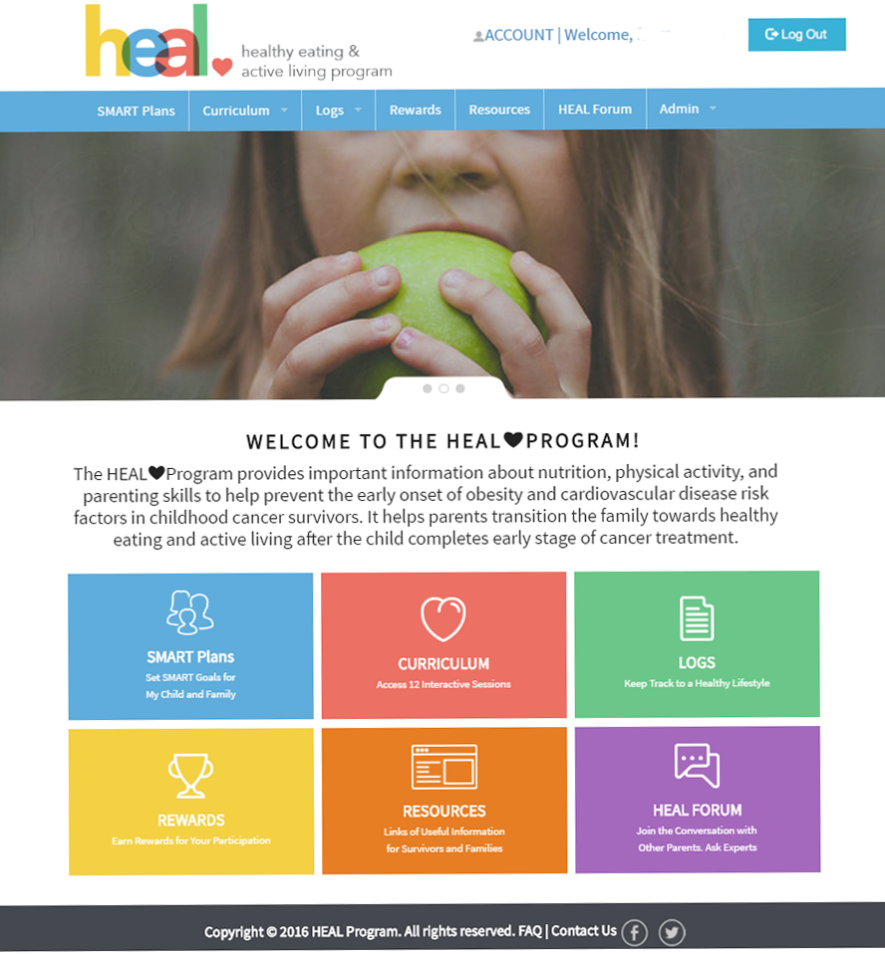
Screenshot of the main Healthy Eating and Active Living component page after log-in.

The HEAL program website was created by an in-house facility, the Center for Engineering Education and Outreach, at Tufts University between January and June 2015. The website has incorporated 4 active modules: Curriculum, SMART Plans, Logs, and Rewards (Figure 3). Additional modules such as Resources and HEAL Forum are optional for providing additional resources when needed. Parents can access the Web-based program from any browser on a computer or mobile phone after registering a HEAL account with a username and password.

**Figure 4:**
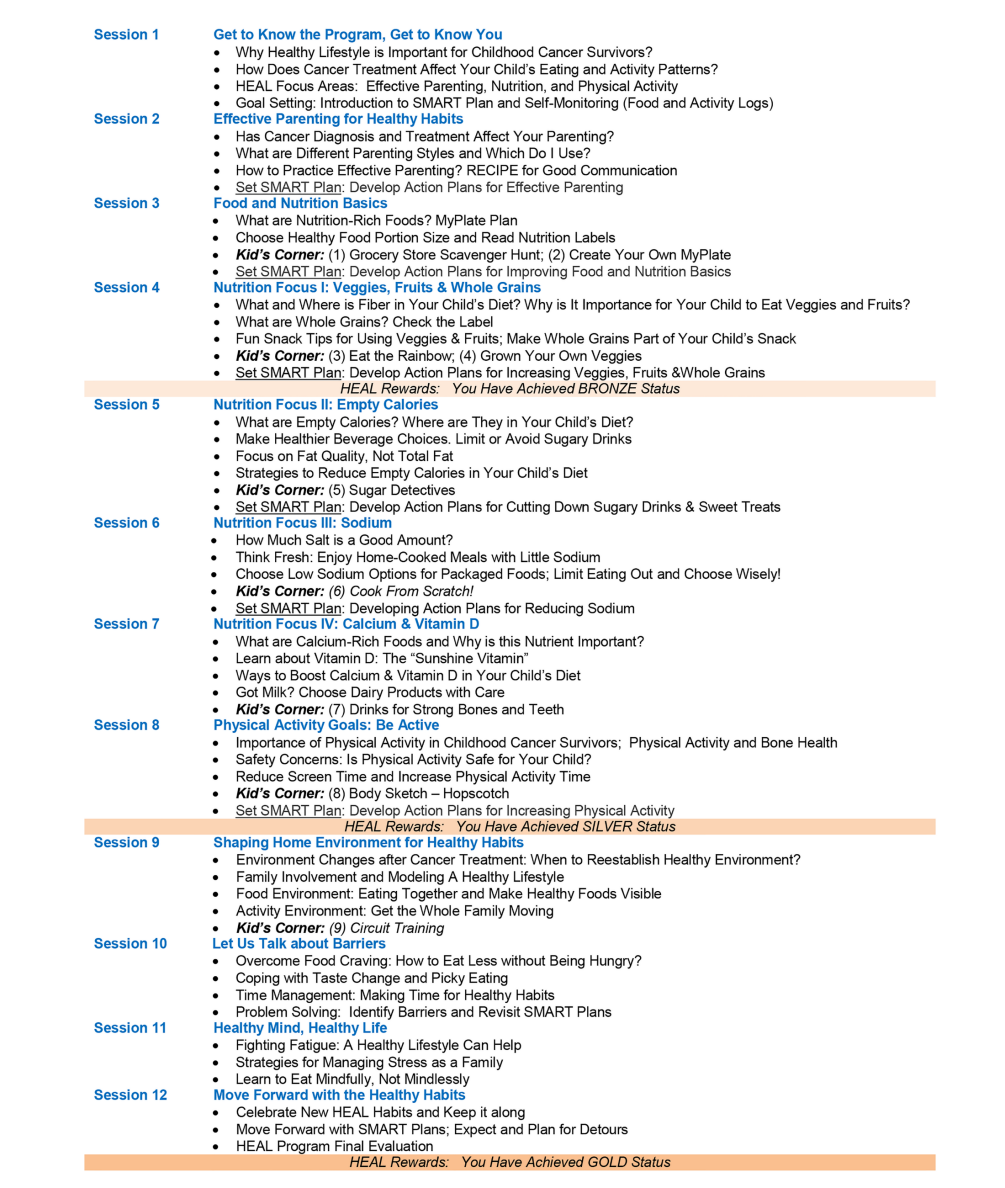
Healthy Eating and Active Living program 12-week curriculum.

The curriculum includes 12 weekly sessions outlining topics on targeted behaviors and specific barriers (Figure 4). Each session takes approximately 30 minutes to complete. The curriculum was initially developed in the written format and went under revision following initial development. The revision was made by the research team based on findings from 4 focus groups conducted with 19 oncology care team members who were invited to provide their perceptions on the content, preferred delivery mode, and timing of weight management for pediatric ALL survivors (detailed results of the focus groups will be provided elsewhere). Specifically, the revision expanded the curriculum by including an audio format for each session created using Articulate to meet the needs of families with low literacy levels. Parents can access either format after they log into the curriculum, but they can only be accessed at the program’s website (ie, not downloadable) so that the program can track usage. In addition, the curriculum contains expanded sessions on specific nutritional issues identified in childhood cancer survivors (eg, high intake of empty calories and sodium and low intake of fiber, whole grains, and vitamin D and calcium) and barriers experienced by the survivors (eg, food craving, change in taste preference, fatigue, stress, lack of time). The revision also includes a module called Kid’s Corner embedded into each individual sessions (see Figure 4) for parents to involve children participating in activities that promote healthy eating (such as *Grocery Store Scavenger Hunt*, *Create Your Own MyPlate*, *Eat the Rainbow*, *Grow Your Own Veggies*, *Sugar Detectives*, *Cook from Scratch*, *Drinks for Strong Bones and Teeth*) and physical activity (such as *Body Sketch-Hopscotch* and *Fun and Fast Circuit Course*) for the whole family.

Parents enrolled in the HEAL program are asked to complete the weekly self-guided curriculum; set up behavioral goals on parenting, nutrition, and physical activity using SMART plans; and complete weekly food and activity logs online (for at least 2 weekdays and 1 weekend per week) for 12 weeks. All curriculum sessions are accessible by the participants after they log in, although instructions are provided in the first session that they are expected to complete one session per week. The HEAL program has built-in functions that monitor how many minutes a HEAL user spends daily on each session to track usage. Based on the usage, the program sends automated motivational messages to parents after completion of goal-setting, logs, and curriculum session. Parents achieve Bronze, Silver, and Gold status after completing each of the 4 sessions as rewards, and children receive a digital certificate after completing family activities. At the end of each curriculum session, parents are encouraged to complete an online evaluation that asks them to rate statements about their knowledge of session-specific topics before and after each session on a 5-point Likert scale and answer open-ended questions on their perceptions of session strengths and weakness. As part of the study end assessment, parents are asked to complete a usability assessment once they have finished the program.

## Discussion

Childhood cancer survivors are at substantially increased risk of developing cardiometabolic conditions at a young age. Although treatment exposure, alone or in combination, contributes to elevated CVD risk in childhood cancer survivors, the attributable fraction was less than 50%, ranging from 9.3% for hypertension, 15.5% for dyslipidemia, 41.7% for diabetes, and 42.1% for obesity [2]. A large proportion of the CVD burden can be reduced through lifestyle interventions. Nevertheless, promotion of healthy eating, active living, and weight management is not routinely integrated into cancer care for childhood cancer survivors and families in many oncology clinics around the country. The HEAL program addresses this gap by empowering parents with knowledge and skills to facilitate healthy eating and active living as soon as the child completes intensive cancer treatment and is ready for transition. To our best knowledge, it is among the first to introduce the importance of healthy eating and active living early in cancer care (during maintenance therapy or within 2 years of treatment completion) in order to capture a sensitive window of unhealthy weight gain and prevent the early onset of CVD risk factors in childhood ALL survivors.

Many survivorship programs for childhood cancer lack a focus on nutrition, or when nutrition is introduced, the priority is to satisfy caloric needs and prevent weight loss. Although malnutrition due to cancer-related anorexia and cachexia still represents an important concern in cancer care [33], overconsumption of calories through poor eating habits [12,15-17] and a high prevalence of obesity are increasingly recognized in childhood ALL survivors [2,34]. Weight management programs for childhood cancer survivors should respond to the growing need to curb early onset of obesity and CVD risk factors by reducing overconsumption of calories to achieve energy balance. It is also important to note that maintaining energy balance should focus on improving diet quality and healthy eating patterns rather than on calorie restriction alone [35]. The nutrition component of weight management must address survivors’ targeted nutritional needs such as excessive intakes of empty calories and sodium and inadequate intakes of vegetables and whole grains identified in the survivors [12,15-17] in order to be effective. These nutritional targets are similar to those identified in the general population. However, childhood cancer survivors experience a substantially higher CVD burden than the general population. Interventions to improve these nutritional targets for a high-risk population can have a much larger impact on reducing the CVD burden than that in the general population. Similarly, a large proportion of childhood cancer survivors have low bone mineral density due to exposure to glucocorticoids treatment [30,31]. Having adequate vitamin D and calcium intake from dietary sources and increasing bone-strengthening activities are important targets for improving bone health in this population. It is equally important to recognize that cancer treatment can have a long-lasting impact on survivors’ eating patterns or activity levels, such as food craving [36], change in taste preference [37], fatigue [38,39], and stress [40]. These barriers may prevent childhood cancer survivors from making healthy food choices and being physical activity. Therefore, the weight management program for childhood cancer survivors should meet their targeted nutrition needs and help survivors and parents identify options to overcome barriers.

Parenting styles and practices are important behavioral targets for weight management in childhood cancer survivors. For young survivors (aged 10 years or under) with limited autonomy and greater dependence on caregivers, family environment plays a highly influential role in facilitating a child’s eating and activity behaviors. For families with childhood cancer survivors, family environment can be even more important, because a close parent-child relationship is often expected in this context [41]. Qualitative research suggests that parents tend to change parenting styles after a child’s diagnosis and treatment of cancer [26]. Parents may practice permissive parenting, allowing their child to choose highly preferred, processed snack foods. Parents may also practice protective parenting, encouraging sedentary behavior because of concerns about the safety of exercise. Prior research provides convincing evidence that interventions targeting parents exclusively or targeting parents and children together yields greater success in preventing childhood obesity than those targeting children alone [42-44]. Therefore, weight management programs should engage parents as the agents of change, improve parenting style and practices, and emphasize the parents’ roles in transitioning the family towards healthy eating and exercise through modifying family environment. Since these types of programs are more effective when based on behavioral theory [45], a strong theoretical framework underpins the HEAL program. A limitation of the program, however, is that it does not explicitly address the family structure of the intervention. Although the program can be accessible by any parent or caregiver after he or she registers for an account, it does not address behavioral issues that may arise when both parents or all caregivers enroll in the program.

An important characteristic of childhood cancer survivors is that they are geographically dispersed. As a consequence, they have transportation difficulties coming to interventions delivered at a central location. Intensive time commitment to cancer-related care adds an additional barrier for families to participate in interventions scheduled at fixed times [46]. On the other hand, 87% of the American adults have Internet access and 90% have a mobile phone [47,48]. Access to Internet and mobile phones is observed across all major ethnic groups and socioeconomic backgrounds. Interventions delivered remotely through Web- and mobile-based apps, combined with individualized feedback and motivational messages provided through emails and phones, can substantially increase reach and reduce cost. In addition, they provide a cost-effective option for integrating weight management into the survivorship care for childhood cancer survivors and families [49].

In summary, lifestyle interventions are becoming a priority in cancer care to prevent excessive weight gain and early onset of CVD risk factors in childhood cancer survivors. These interventions must meet the targeted behavioral needs of the survivors; address specific barriers; capture a sensitive window for behavioral change; and be convenient, cost-effective, and scalable. Future research is needed to evaluate the feasibility of engaging pediatric ALL survivors and families in early weight management either within or outside cancer clinics and to assess participation, adherence, and retention rates. Such findings will provide an important foundation to inform the design and implementation of a fully-powered randomized controlled trial to evaluate the effect of early weight management on preventing unhealthy weight gain and CVD risk in young survivors of childhood ALL and to identify effective strategies to integrate weight management into cancer care and adapt the program to survivors with other cancer diagnoses.

## Abbreviations

ALL: childhood acute lymphoblastic leukemia
BMI: body mass index
CVD: cardiovascular disease
HEAL: Healthy Eating and Active Living
SDT: self-determination theory
SMART: Specific, Measurable, Achievable, Relevant, and Time-bound
SSB: sugar-sweetened beverage
